# Temperature Influence on Ordinary Concrete Modified with Fly Ashes from Thermally Conversed Municipal Sewage Sludge Strength Parameters

**DOI:** 10.3390/ma13225259

**Published:** 2020-11-20

**Authors:** Gabriela Rutkowska, Paweł Ogrodnik, Joanna Fronczyk, Ayla Bilgin

**Affiliations:** 1 Institute of Civil Engineering, Warsaw University of Life Sciences—SGGW, Nowoursynowska 166, 02-787 Warsaw, Poland; gabriela_rutkowska@sggw.edu.pl; 2 Institute of Security Engineering, Main School of Fire Service, 01-629 Warsaw, Poland; pogrodnik@sgsp.edu.pl; 3 Faculty of Engineering, Seyitler Campus, Artvin Coruh University, 08000 Artvin, Turkey; ayla.bilgin@artvin.edu.tr

**Keywords:** concrete, fly ashes, temperature, compressive strength

## Abstract

Concrete is the most commonly used structural material, without which modern construction could not function. It is a material with a high potential to adapt to specific operating conditions. The use of this potential is made by its material modification. The aim of the performed investigations was the assessment of rational application possibilities of fly ashes from thermally conversed municipal sewage sludge as an alternative concrete admixture. A concrete mix was designed, based on the Portland cement CEM I 42.5R and containing various quantity of ash, amounting to 0–25% of cement mass. The samples were conditioned and heated in a furnace at the temperature of 300 °C, 500 °C, and 700 °C. Physical and chemical properties of the ashes as well as utility properties of the concrete, i.e., density, compressive strength after 28, 56, and 90 days of maturation, frost resistance, and compressive strength in high temperature were determined. The tests were performed at cubic samples with 10 cm edge. The replacement of a determined cement quantity by the fly ashes enables obtaining a concrete composite having good strength parameters. The concrete modified by the fly ashes constituting 20% of the cement mass achieved its average compressive strength after 28 days of maturation equal to 50.12 MPa, after 56 days 50.61 MPa and after 90 days 50.80 MPa. The temperature growth weakens the composite structure. The obtained results confirm the possibility of waste recycling in the form of fly ashes as a cement substitute in concrete manufacturing.

## 1. Introduction

The current legislation imposes increasingly complex requirements on building materials. It is aimed at safety, durability, and ecological friendliness of buildings. In the document Model Code 2010 [1], addressed to all scientists and standardizing committees, one of the topics raised is the problem of the environmental impact of constructions, including those made of concrete, as well as issues concerning sustainable development. Simultaneously, management questions of wastes as a resource as well as questions concerning the so-called closed-loop economy are increasingly raised as reflected inter alia (i.a.) in the European Union message [2]. The ecological awareness of the society and, first of all, necessity to base state economies on sustainable development means the question of environmental impact is more and more frequently raised [3,4]. Numerous investigations are undertaken, aimed at the reduction of energy consumption of solutions in the building industry and reduction of wastes produced quantity [5,6].

Portland cement manufacturing technology is a process with a lot of disadvantages, comprising the consumption of a big quantity of natural resources, very high energy consumption, and significant environmental pollution, including high emission of CO_2_ resulting from the decarbonization of raw material [7,8,9,10]. The significant energy consumption is due to a high temperature needed to run the clinker burning process. The Portland cement manufacturing requires the temperatures 1400–1500 °C. Limitations of the emission of CO_2_, imposed by the European Union [11], prompt researches on innovative materials containing lower quantities of clinker.

Nowadays, calcareous and siliceous fly ashes originating from the combustion of hard and brown coal are widely applied in concrete manufacturing technology [12]. The crucial parameters of the fly ash, which determine its application in concrete manufacturing technology, are: chemical and phase composition, high fineness close to that of cement, as well as reactivity and, above all, pozzolanic activity.

Per the requirements for quality control procedures, the chemical and physical properties for fly ash used as a type II additive in the concrete production are specified in PN-EN 450-1:2012 norm [13,14,15]. In Naik, Kraus and Siddique research [16], the influence of the wood fly ash on the concrete frost resistance was investigated. Three mixes were tested with assumption that 5%, 8%, and 12% of the bond mass was replaced by the fly ashes. Making such assumptions, no significant changes in the application of organic fly ashes, i.a., from the combustion of rice hulls or wood, in concrete manufacturing is still developing. The research direction of the frost resistance of samples in relation to the control sample as well as no changes of the elasticity modulus were observed. In Teixeira, Camões and Branco research [17], a review of research concerning application of the wood fly ash (WFA) as a cement substitute was made. The results showed that increase of the WFA quantity negatively affects concrete features compared to the conventional concrete. The application of such ashes brings similar results as it is for the coal ashes. It was observed that, in most cases, the fly ash admixture concretes present higher strength and durability. It is also confirmed by the results of investigations presented in [18,19,20]. Nowadays, worldwide, commonly on an industrial scale, ashes are applied, which come as a result of hard and brown coal combustion [21,22,23]. Their application is mainly determined by mineral and chemical composition, as well as the pozzolanic activity index, similar to cement [24,25]. The fly ashes from the biomass combustion are also widely used. In the most recent investigations [26,27], it has been confirmed that the application of FBC (fly ash from fluidized bed combustion) in the concrete can increase resistance against the penetration of chloride ions with a simultaneous maintenance of comparable strength. In the investigations [28], the Portland cement 42.5R was replaced by the fly ashes from the combustion of biomass in quantities in the range of 5%–30%. The authors performed researches on a large scale, including strength tests (compressive strength), frost resistance tests, and absorbability tests, simultaneously determining such features as density and porosity. It was proven that fly ashes application amounting to 15% of the cement mass evoked an increase of the compressive strength after 28 days of maturation accompanied by lower absorbability and higher frost resistance.

Sewage sludge produced in sewage treatment plants are a big environmental problem. They are often utilized with the use of incineration, which produces a fly ash. The main goal of the conducted experimental work is to examine the possibility of using ashes from sewage sludge in the concrete production [29,30,31,32,33]. Their pozzolanic activity, which has been confirmed in the investigations [30,34] along with other physical and chemical properties, makes them an admixture with big application potential. Their application in the concrete receipt contributes to savings in mineral fuels and natural raw materials, and also reduces environmental pollution and the emission of CO_2_ [35,36,37]. In recent years, the number of municipal sewage sludge incineration plants has increased. Their total processing capacity amounts to 160,300 Mg [38] of dry mass per year. As a thermal combustion result of sewage sludge, significant quantities of fly ashes are produced, which are classified as a waste with code 19 01 14 [39], and they must be appropriately managed. According to the directive of the European Parliament and Council [40], the resulting fly ash from combustion can be used to prepare a concrete mix for construction purposes, without facilities for people or animals with permanent residence, and for food storage. The research to-date that points to the application of fly ashes from thermally converted municipal sewage sludge allows to achieve comparable properties in relation to a concrete manufactured on the basis of the widely used fly ashes (siliceous and calcareous fly ashes) [37,41,42,43]. The investigations [44,45] considered the replacement of 20% of cement by the fly ashes from thermally converted municipal sewage sludge and showed a reduction of the concrete strength within the range of 25–50%. In another investigation [46], 20% of the aluminosilicate metakaolin was replaced by the fly ashes from thermally conversed municipal sewage sludge. Mortars hardened in the temperature 65 °C presented strength reduction with relation to those hardened in the temperature 25 °C. Similar results concerning the reduction of bending and the compression strength of mortars without admixtures were obtained in [47]. Some researchers, however, point to a slight increase of strength of the samples containing fly ashes from thermally conversed municipal sewage sludge [48,49,50]. In these investigations, the authors applied fly ashes from thermally converted municipal sewage sludge in the amount of 15–20% of the cement mass, achieving compressive and bending strength 8–15% higher than that of control samples. These results should be not surprising due to the different physical and chemical composition of the fly ashes from municipal sewage sludge, which can affect concrete properties.

Destructive changes in the concrete composites containing the Portland cement are evoked by high temperatures that occur during fire. In previous studies [51], it has been shown that, along with the temperature growth, the compressive strengths of concrete reduces by several tens of percent. A High temperature behavior research of concretes containing fly ashes has been also conducted [52]. It presents a positive influence of the fly ashes replacing 25% of e cement mass on the carbonatization depth in the concretes subjected to thermal loads within the range 150–600 °C. Similar results were obtained for investigations [53] performed for the temperatures of 150–550 °C. It was also shown [54] that the fly ash positively affects the strength of the concrete subjected to a thermal processing.

This paper is a continuation of the authors previous works, and aside from concrete strength features with various content of fly ashes (FA) from thermally converted municipal sewage sludge, it presents their frost and high temperature resistance. The aim of experimental research was to evaluate the effect of temperature on frost resistance strength parameters of concretes produced with the use of fly ash from the thermal treatment of sewage sludge. The article also presents tests results and analyses of fly ash from sewage sludge properties.

## 2. Materials and Methods

### 2.1. Preparation of Concrete Specimens

In aim to perform investigations, an ordinary concrete mix of class C20/25 and consistency S3 was designed with the use of a calculation and experimental method based on the Bukowski’s three equations method. The main components of concrete mix are: Portland cement CEM I 42.5R from Cement Ożarów S.A. (Ożarów, Poland), sand aggregate of 0–2 mm fraction, gravel aggregate 2–16 mm fraction, fly ashes from thermally conversed sewage sludge from Cracow, and water. In all samples, the same granulometric composition of the fine aggregate (0–2 mm), selected with use of the sieve analysis, and the same composition was assumed of coarse aggregate selected with subsequent iterations [55] (Table 1). The chemical and physical parameters as well as the phase composition of the cement according to the standard PN-EN 197-1:2012 [56] are presented in Table 2 and Table 3 As a partial cement substitute, a mineral admixture was used, composed of the fly ashes from thermally conversed municipal sewage sludge from the sewage treatment plant in Cracow.

With the aim to perform a comparative analysis of properties of the ordinary concrete and concretes containing the fly ashes from thermally converted municipal sewage sludge, mixes were prepared with various contents of admixture (0%, 2.5%, 5%, 7.5%, 10%, 12.5%, 15%, 17.5%, and 20%), as shown in Table 4. The reference mix was modified by the fly ashes, with the simultaneous optimization of water and cement quantity with consideration of the contents exchanged volume.

The concrete mix consistency was checked with use of concrete slump test (PN-EN 12350-2:2011, [57]), the bulk density, with use of mass and volume measurements (PN-EN 12350-6:2011 [58]), and the air content test, with use of pressure method (PN-EN 12350-7:2011 [59]). For experimental investigations, cubic samples with dimensions of 100 × 100 × 100 mm were prepared and stored in a bath according to the standard PN-EN 12390-3:2019 [60]. The samples were tested in respect of selected properties: compressive strength, high temperature resistance, frost resistance. The investigations were performed in the Warsaw University of Life Sciences in the Institute of Civil Engineering, as well as in the Main School of Fire Service in Warsaw.

The compressive strength tests were performed according to PN-EN 12390-3:2019 directives, after 28, 56 and 90 days of maturation [60]. The tests were performed in the H011 Matest hydraulic testing machine (Matest, Brembate Sopra, Italy. Basing on the average values of strength, the result was recalculated into cubic samples with an edge of 15 cm (pattern) and the concrete class was established:

f_c,cub150_ = 0.90 f_c,cub150_ (1)

where f_c,cub150_, f_c,cub100_ denotes the compressive strength observed on cubic samples with the edge length equal to 15 and 10 cm, respectively.

The frost resistance tests were performed with use of a direct method according to the procedure described in the standard PN-88/B-06265 in the Toropol cold chamber [61]. In the statistical analysis, for the assumed number of 6 samples, the statistical parameters were determined: standard deviation, variability coefficient and total uncertainty for the recommended confidence interval at the level *p* = 0.95. With the aim to assess the expanded (total) uncertainty, the sclerometric unscaled method was used [62,63].

### 2.2. High Temperature Tests of Concrete

The thermal resistance of the composite against high temperature was investigated in a special furnace PK 1100/5 from Termolab S.C. (Warsaw, Poland). The samples were held in the temperature 300, 500 and 700 °C. The furnace had electrically supplied heating sections. The rig is equipped in the dedicated program ThermoPro (1.0, Thermolab S.C., Warsaw, Poland) which enables programming of heating process. The temperature distribution in the tested element was monitored with use of the thermocouples NiCr-Ni, fulfilling the standard requirements [64].

**Figure 1:**
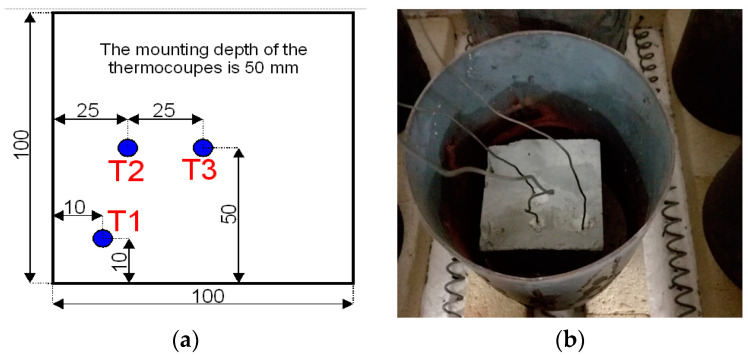
View of cubic sample: (**a**) Layout of thermocouples; (**b**) View of a sample with measuring thermocouples.

Before the start of the heating process, all samples had been dried out to a constant mass at the temperature of 105 °C ± 5 °C. The signal from the thermocouples was recorded by a computer equipped with the dedicated software. The tests were performed according to a standard curve temperature vs. time. With the aim to determine time of holding in individual temperatures, specifically prepared samples with borings for measuring thermocouples were subjected to pilot tests. The temperature distribution was monitored by 4 thermocouples (Figure 1). The borings for the thermocouples were made in the middle of the sample (T3), 25 mm from the base edge (T2) and 10 mm from the base edge (T1). The thermocouples were introduced through the rear wall and placed near to the furnace ceiling. The borings’ depth was equal to 50 mm. The tests took selected parameters of fire surroundings into account and a forecasted heat process was programmed. The thermal conditions of fire in the furnace chamber were described by a standard curve temperature vs. time [65]. The investigations performed during holding of the samples correspond to the conditions existing in fire [66].

### 2.3. Characteristics of Fly Ashes from Thermally Conversed Sewage Sludge

Aiming to recognize fly ashes tested, the following characteristics were determined: (1) the chemical composition using the energy dispersive X-ray fluorescence (XRF) method; (2) the mineral composition using X-ray phase analysis (XRD); (3) the grain size distribution using a laser diffraction; (4) the morphology and chemical composition using scanning electron microscope technique; and (5) the pozzolanic activity according to the standards [13,67] and requirements contained in related literature [68]. Detailed information on the conditions for conducting the above-mentioned tests, as well as the equipment used, have been presented in previous publications [29,32].

### 2.4. Statistical Analysis

Statistical methods are useful for interpretation and modeling of large data sets. In this study, variance analysis (ANOVA) was used. ANOVA analysis is used to determine the difference of more than two independent variables. SPSS program (19, IBM, Armonk, NY, USA) was used to analyze the data.

## 3. Results and Discussion

### 3.1. Properties of Fly Ash and Concrete Mix

#### 3.1.1. Fly Ash

**Figure 2:**
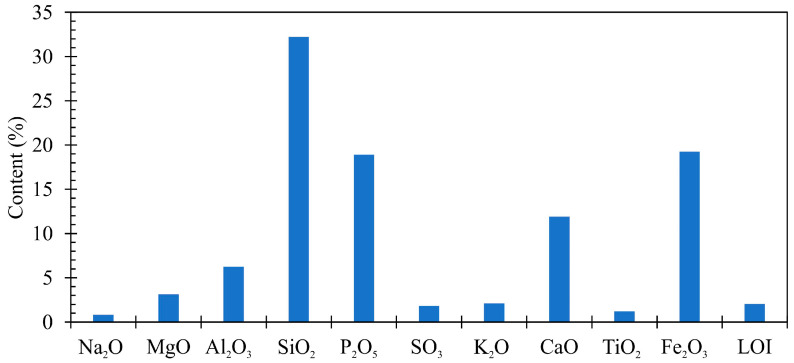
Chemical composition of fly ashes from thermally conversed municipal sewage sludge.

Analysis results of fly ashes from municipal sewage sludge composition are presented in Figure 2. The loss in ignition of fly ashes tested sample was equal 0.56%. This is due to the combustion temperature in a fluidal furnace, exceeding 850 °C. The highest percentage share in the samples concerned the silica (32.21%), iron oxides (19.25%) and phosphor oxides (18.91%). Moreover, the total content (57.71%) of the silica (SiO_2_ 32.21%), aluminum dioxide (Al_2_O_3_ 6.25%), and iron oxide (Fe_2_O_3_ 19.25%) in the sewage sludge fly ashes did not satisfy the requirements contained in the standard PN-EN 450-1+A1:2012 and concerning siliceous fly ashes (min. 65%) [13]. The grain density according to the standard PN-EN 1097-7:2008 [69] was equal to 2780 kg/m^3^, the fineness according to the standard PN-EN 451-2:2017-06 [70] 46.2%, the volume constancy according to the standard PN-EN 451-1:2012 [13] and PN-EN 196-3:2016-12 [71] 0.5 mm. It was observed that the fly ash used from the thermal treatment of sewage sludge from the sewage treatment plant in Kraków was characterized by a better chemical composition than the ash used by the authors [32] from the sewage treatment plant in Warsaw. The content sum of silicon dioxide (SiO_2_-17.8), aluminum oxide (Al_2_O_3_-11.1), and iron oxide (Fe_2_O_3_-6.5) in these ashes was lower and amounted to only 35.4.

**Figure 3:**
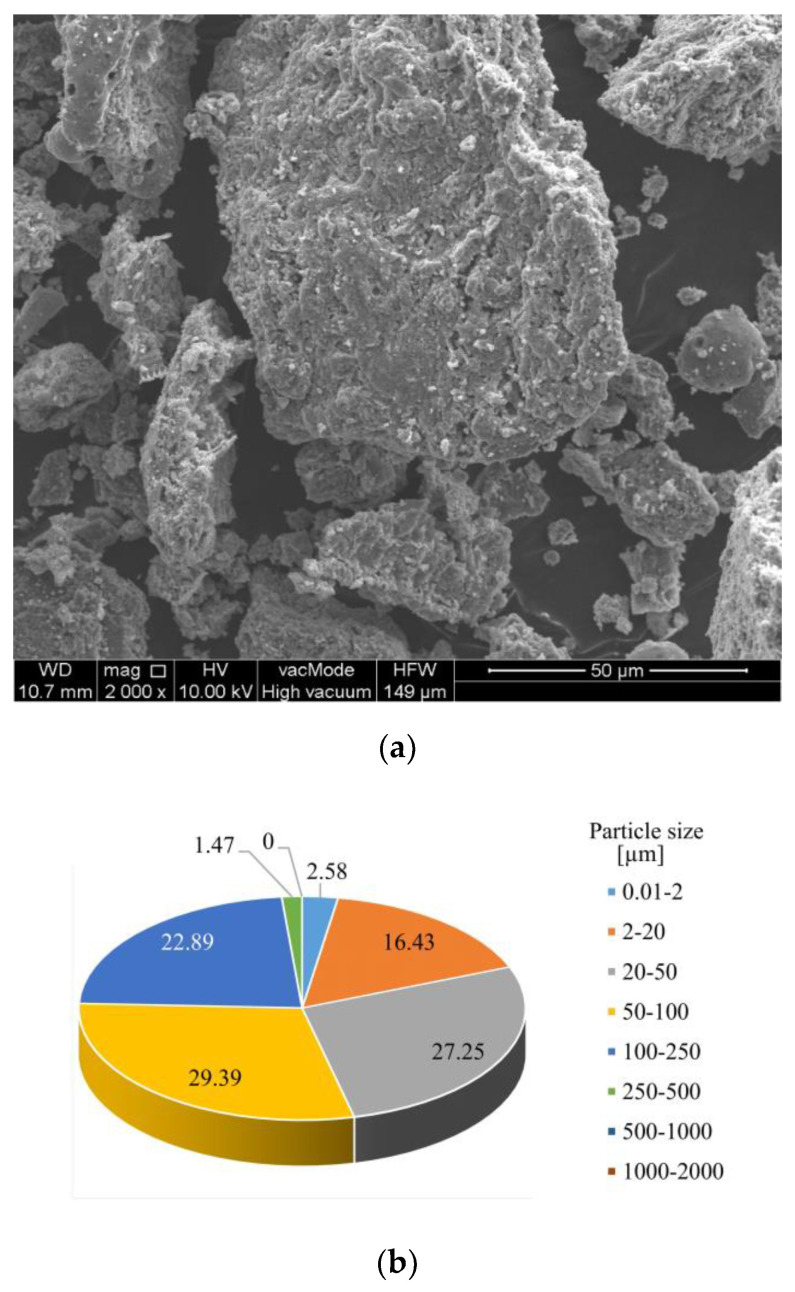
SEM image (**a**) and volumetric distribution of individual fractions in the tested fly ashes (**b**).

Figure 3 presents scanning electron microscope (SEM) images and volumetric distribution of individual fractions of the fly ashes from thermally conversed sewage sludge. The dominating grain fractions are 20–50 µm (27.25% vol.), 50–100 µm (29.39% vol.), and 100–250 µm (22.89% vol.).

It was observed that the fly ash samples were dominated by irregular grains with variable size with highly porous, strongly developed surface as well as loose and rough structure. Spherical and cuboidal forms were very rare.

**Figure 4:**
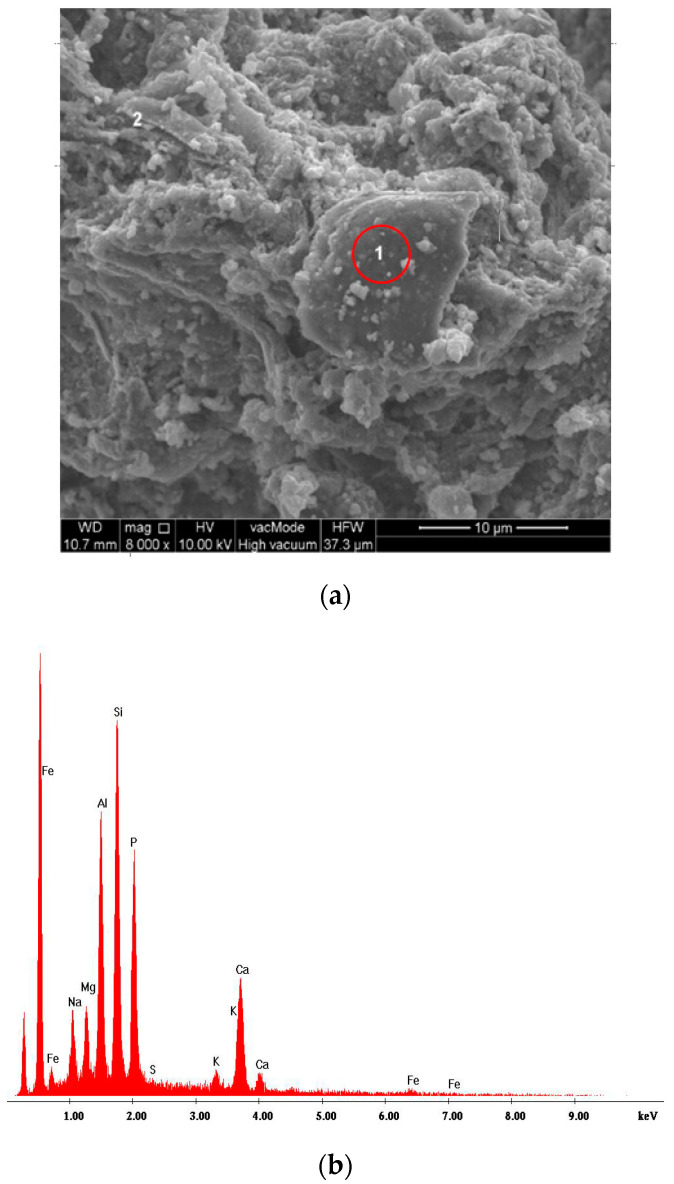
SEM images (**a**) of the tested fly ashes with EDS analysis of the point marked as “1” (**b**).

**Figure 5:**
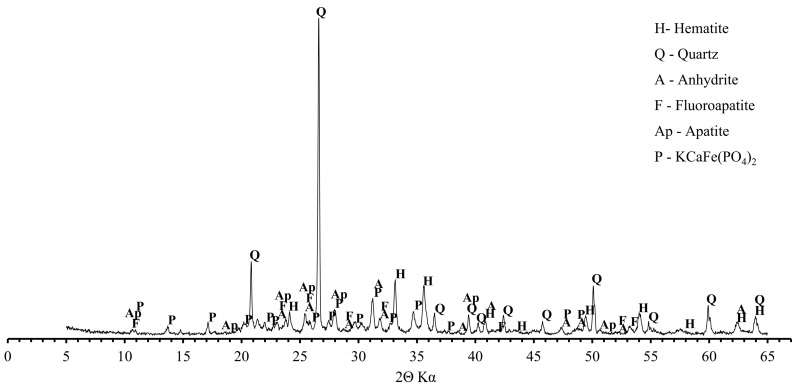
XRD pattern of fly ashes from sewage sludge combustion.

The SEM-EDS chemical microanalysis proved the domination of the silicon, aluminum, phosphorus, and iron grains (Figure 4). Such components as calcium, potassium, and sulfur were present in small quantities. XRD pattern of the fly ashes from sewage sludge combustion is shown in Figure 5. The mineral composition of the fly ashes is dominated by hematite, quartz, and anhydrite. They are supplemented by phosphates in fluorapatite and apatite form.

The fly ashes demonstrate the pozzolanic activity, i.e., they react with calcium hydroxide in its presence (it can originate from hydrolysis of klinker cement siliceous phases), creating products having hydraulic and binding properties [72,73,74,75]. The reactivity of fly ashes with Ca(OH)_2_ is affected by their chemical and mineral composition. The performed investigations of fly ashes from thermally conversed sewage sludge pozzolanic activity allowed to assess the application possibilities of this admixture in concrete modification. According to the standard ASTM C379-65T [67], the pozzolanic activity is defined as the total content of the reactive SiO_2_ and Al_2_O_3_. The pozzolanic activity, established in the performed investigations, was equal to 20.49%. The total content of the aforementioned oxides over 20% indicates a pozzolanic character [76,77]. According to the standard PN-EN 450-1:2012 [13], the fly ash quality is determined by the activity index as the compressive strength ratio of a standard mortar prepared with use of the Portland cement to the compressive strength of a mortar containing 75% of cement and 25% of fly ashes, determined after 28 and 90 days of maturation. The time 28 days is related to the curing time, and 90 days to the time of free reaction of active components of the admixture with the calcium hydroxide. The activity index after 28 days of maturation should exceed the value 75% and, after 90 days, 85%. The activity index of the applied fly ashes from thermally converted sewage sludge was equal 68.9% after 28 days of maturation and 78.9% after 90 days of maturation. However, this standard concerns siliceous fly ashes produced during coal combustion or co-combustion with waste. The ashes from the burning of sludge from Warsaw showed a higher pozzolanic activity at the level of 92%, which allows them to qualify as active mineral additives [32].

According to Hubbard and Dhir [68], the fly ash activity is determined by the ratio of potassium oxide to calcium oxide (K_2_O/Al_2_O_3_). The so-called desirable pozzolanic potential, introduced by these authors, classifies fly ashes into three classes depending on this oxide ratio: 1st class-K_2_O/Al_2_O_3 ·_10 ≥ 1, 2nd class-0.5 < K_2_O/Al_2_O_3 ·_10 < 1, 3rd class-K_2_O/Al_2_O_3 ·_10 < 0.5. For the investigated fly ash, a result 0.34 was obtained which categorizes the fly ash as little reactive-class 3.

#### 3.1.2. Concrete Mix

**Figure 6:**
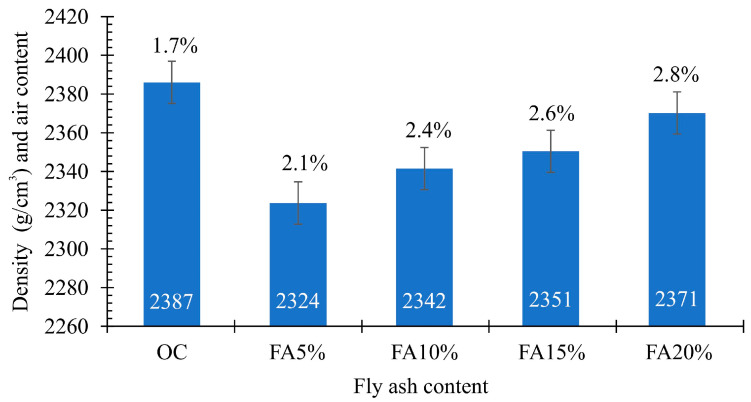
Air content and density of the concrete mixes.

Basing on the performed investigations, it has been stated that the consistency determined in the concrete slump test can be classified as plastic for the reference concrete mix and for all samples containing various quantities of the fly ashes from thermally conversed sewage sludge. The density of the concrete mix reached values falling into the range 2387 (OC)-2371 kg/m^3^ (FA20%). The smallest air amount was observed in the concrete mix of reference concrete OC, equal to 1.7%, while the highest, equal to 2.8% was in the mix FA20% (Figure 6).

### 3.2. Properties of Concrete

#### 3.2.1. Compressive Strength

**Figure 7:**
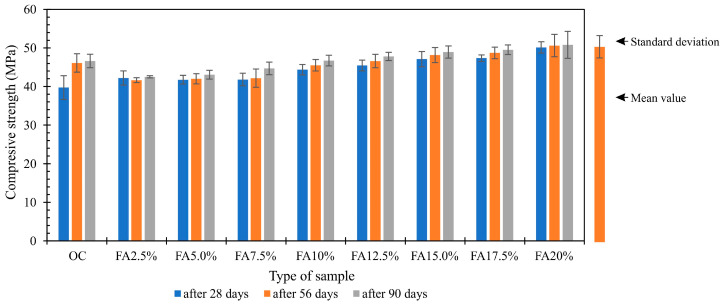
Compressive strength after 28, 56 and 90 days of maturation.

Results of s of average compressive strength measurement of concrete samples with various content of the fly ashes from thermally conversed sewage sludge is presented in Figure 7.

After the first maturing period, after 28 days, it was observed that the lowest compressive strength of 39.74 MPa was achieved by concrete FA2.5%, while the highest, equal to 50.12 MPa, was obtained by concrete FA20%, in which cement was replaced with ash from sewage sludge in the amount of 20%. Compared to the reference concrete without additive, the decrease in compressive strength of concrete FA2.5% was 5.7% and the increase in concrete FA20%–18.7%. The highest compressive strength after 56, equal to 50.61 MPa and after 90 days equal to 50.80 MPa, was also achieved by concrete FA20%, the lowest strength after 56 days equal to 41.64 MPa and after 90 days equal to 42.52 MPa concrete FA2.5%. After the first compressive strength measurement, i.e., after 28 courses of curing, it was observed that the reference concrete (OC) achieved 90.4% of the final strength (after 90 courses of curing). Concretes modified with fly ash from thermal treatment of sewage sludge reached values f_cm_ within the range of 93.4% (FA2.5%) – 98.7% (FA20%). Replacing cement with ash from sewage sludge in an amount greater than 10% causes an increase in strength in relation to the reference concrete without the addition (Figure 7). The research conducted by the authors [32,33,77] on the effect of fly ash from thermal transformation of sewage sludge confirms the positive effect on modified concretes strength properties. The chemical composition of the fly ashes (silica, calcium, magnesium, iron) and their pozzolanic properties indicate the application possibility of this admixture in the concrete manufacturing technology, like the traditional mineral admixtures [42,78]. It has been stated in the investigations [33,76] as well that if over 15% of cement is replaced by the fly, it affects the mortar strength. According to the results presented by other authors, the maximum exchange of the cement to the fly ashes amounts 5–20% [79,80,81]. Taking into account the chemical composition of the fly ash used [32], it was noticed that higher compressive strength values were obtained for concrete in which fly ash from Kraków was used than for concrete with ash from Warsaw. The lower concentration of P_2_O_5_, CaO, SiO_2_, and Al_2_O_3_ compounds and higher SiO_2_, Al_2_O_3_, and Fe_2_O_3_ increase the strength of produced concretes.

The concentration of P_2_O_5,_ CaO, SiO_2_, and Al_2_O_3_ compounds has a fundamental influence on the concrete properties. An increase in the share of these oxides in the composition of fly ash leads to a decrease in compressive strength. On the other hand, the increase in the sum of silicon content, aluminum, and iron oxide in the ash composition increases the compressive strength.

#### 3.2.2. Frost Resistance

The frost resistance test consists in determining the decrease in compressive strength of a sample frozen at a temperature of ± 20 °C in relation to a non-frozen sample. The reduction in compressive strength should not exceed 20%. According to the PN-88/B-06250 [61] standard, the samples subjected to freezing should not have cracks or damages, and their weight loss should not exceed 5%. Table 5 summarizes the set of obtained results of the compressive strength of reference concrete samples (witnesses) and concrete samples after the cycles of freezing and thawing (F150).

Concrete modified with fly ash from sewage sludge up to 15% is frost-resistant concrete F150. The lowest loss in strength was observed for the sample with 2.5% of cement replaced by the ashes (FA2.5%), whereas the highest-for the FA20% samples (with 20% ash content). The values amounted 2.69% and 22.69%, respectively. The average loss in mass was slight in all cases and oscillated between 0.251% (FA15%) and 1.037% (FA5%). The compressive strength of reference concrete samples ranged from 44.62 MPa (for FA2.5%) to 57.25 MPa (for FA20%). After 150 cycles of freezing and thawing at a temperature of ± 20 °C, it was observed that the highest compressive strength equal to 44.56 MPa was achieved by concrete in which 20% of cement was exchanged for fly ash. On the other hand, the lowest strength of 42.26 MPa was achieved by concrete without the addition of CO.

#### 3.2.3. Compressive Strength at High Temperatures

**Figure 8:**
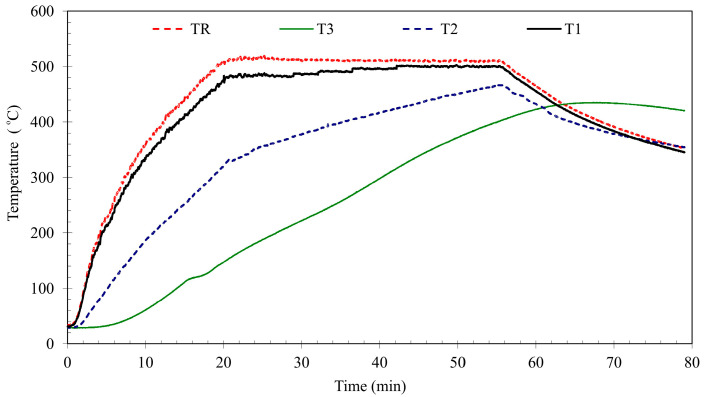
Temperature distribution in the OC pilot sample. Heating curve 500 °C.

A temperature increase weakens a material structure. Cracks and scratches were visible on the sample surface. A real example temperature distribution presented in Figure 8 allows for the observation of concrete in fire behavior.

The thermocouples distributed in the pilot sample allowed modeling of a standardized fire development. A heating process was programmed by setting heating time, temperature and time sections number of the process which had to be realized. The point where temperatures equalize shows that the sample is evenly heated in its volume. In the self-cooling phase temperature increase of external layers is visible what is confirmed in investigations [4].

After holding in the temperature 300 °C, it was observed an increase of the average compressive strength, related to the strength determined for the room temperature, and it was equal to 9.0 MPa for the OC concrete and 2.9 MPa for the FA5% concrete. For the remaining concretes, the compressive strength fell for the FA10% by 3.6MPa, for the FA15% by 8.1 MPa, and for the FA20% by 10.4MPa.

Fire temperatures exceeding 500 °C evoke a decrease in the compressive strength by ca. 11% for the FA5%, 24% for the FA10%, 38% for the FA15%, and 30% for the FA20%. The concretes in the temperature 700 °C reduced their strength in the range between 31% (OC) and 61% (FA15%) related to the samples made of the reference concrete (not heated). A lower content of the fly ashes from thermal conversion of sewage sludge in concretes positively affects the compressive strength of the samples subjected to the action of fire.

In this study, one-way ANOVA analysis was performed to determine the differences between compressive strength, temperature, and fly ash amount. According to ANOVA analysis results, the parameters of OC, FA5%, FA10%, FA15%, and FA20% among different temperature (200 °C, 300 °C, 500 °C, 700 °C) are statistically significant different (*p* < 0.05) (Table 6). A further analysis was performed to identify the compressive strength where these parameters vary. For the OC and FA5% samples, there is a statistical difference for temperatures of 20, 300, and 700 °C, except for 20–500 °C degrees (*p* < 0.95). There is a statistical difference in all temperature measurements for FA10%, FA15%, and FA20%.

ANOVA analysis was also performed to determine the differences between compressive strength of control samples (samples that were not subjected to cyclic freeze-thaw) and after 150 freeze-thaw cycles. According to ANOVA analysis results, the values of compressive strength are statistically significant different (*p* < 0.05) (Table 7). For the control samples, there is a statistical difference between OC and FA2.5%, FA7.5%, FA12.5%, FA15%, and FA20%, except for FA5%, FA10%, and FA17.5% (*p* < 0.95). For the samples tested, after 150 freeze-thaw cycles, there is a statistical difference between OC and FA12.5%, FA17.5%, and FA20%, except for FA2.5%, FA5%, FA7.5%, FA10%, and FA15% (*p* < 0.95).

## 4. Conclusions

From an ecological point of view, the re-use of wastes from the thermal conversion of sewage sludge in the concrete manufacturing technology brings economic advantages. The investigations proved that the fly ash can perfectly substitute cement as a hydraulic mineral binder obtained from mineral raw materials.

After the analysis of the results and observations made during the performed experimental investigations, it can been stated that:

1. There is a possibility of using waste in the form of fly ash from the thermal treatment of sewage sludge as an additive (partial cement substitute) for the concrete production. Fly ash in the amount of up to 20% does not deteriorate the compressive strength and frost resistance.
2. The highest average compressive strength, equal to 50.12 MPa, 50.61 MPa, and 50.80 MPa, after 28, 56, and 90 days of maturation, respectively, was obtained by concrete FA20%, in which 20% of cement was replaced with fly ash from thermal treatment of sewage sludge. The lowest average compressive strength of 39.74 MPa, 41.64 MPa, and 42.52 MPa after 28, 56, and 90 days of maturing was achieved by concrete FA2.5%. All concretes reached the designed (assumed) concrete class C20/25.
3. The chemical and physical composition of the fly ashes from sewage sludge is different than that of fly ashes from the combustion of hard coal and does not fulfill the standard PN-EN 450-1:2012 requirements. The highest percentage share in the fly ash samples corresponded to silicon, calcium, phosphorus, and aluminum oxides. However, there are no regulations concerning the physical and chemical properties of ashes from sewage sludge incineration that would limit the possibilities of their use in concrete technology.
4. The pozzolana activity of the fly ash does not meet the applicable requirements of the PN-EN 450-1:2012 standard after 28 (≥75%) and 90 days (≥85%) of maturation. Fly ash from sewage sludge reaches the required values after a longer maturation period, which allows it to be classified as an active mineral additive.
5. Concrete samples containing in their composition fly ash from incineration of sewage sludge were characterized by a comparable average compressive strength to the comparative concrete without the addition. If the fly ash content does not exceed 20% of the cement mass, the ashes can be applied as a cement substitute.
6. The investigations showed that the concretes containing 2.5–15% of the cement mass are frost-resistant. Concrete made on the basis of fly ash from sewage sludge was characterized by the best values of compressive strength after 150 cycles of freezing and thawing. A slight strength increase was observed as the content of fly ashes from municipal sewage sludge rose within the range 2.5–15% of the cement mass.
7. After heating the concrete samples to the temperature of 300 °C, a compressive strength increase was observed by 3 MPa for the FA5% concrete and by 9 MPa for the OC concrete. The temperature increase to 700 °C caused the compressive strength to decrease in the range between 31% (OC) and 61% (FA15%).
8. During annealing at the temperatures 300–700 °C, the samples did not experience any thermal chipping, which is undoubtedly beneficial due to its destructive and dangerous course.
9. The temperature influence caused the structure damage of tested concrete surface, visible scratches appeared on the outer surfaces. This phenomenon is common due to Portland cement use.

The concrete composite modified with the waste product from the thermal conversion of sewage sludge is characterized by satisfying usable parameters, which was confirmed by the performed experiment.

It is difficult to find in sources any information concerning the influence of the fly ashes from the thermal conversion of sewage sludge on the strength parameters and frost resistance of concrete composites. Information concerning the resistance of these concretes against high temperature is also limited. The facts presented in this paper constitute a basis for further investigations within this framework. Due to regulations and lack of and standards concerning the application of fly ashes from the thermal conversion of sewage sludge, any further interpretation of detailed results requires supplementary investigations.

## Figures and Tables

**Table 1:** Percentage contain of the aggregates selected by iterations.

Fraction	Fraction Mixing Percentage Ratio (for Sand and Gravel)			Grain Composition	
	I Stage	II Stage	III Stage	Sand	Gravel
0.0–0.125	-		31	1.93	0.60
0.0125–0.25				17.82	5.52
0.025–0.50				28.62	8.87
0.50–1.0				24.32	7.54
1.0–2.0				27.31	8.47
2.0–4.0		35	69	-	24.15
4.0–8.0	45	65		-	20.18
8.0–16.0	55			-	24.67

**Table 2:** Physical properties and phase composition of cement CEM I 42.5 R.

Blaine Specific Surface Area (cm^2^/g)	Beginning of Binding Time (min)	Compressive Strength after 2 days (MPa)	Compressive Strength after 28 days (MPa)
3330	218	21.0	49.8
**Share of Mineral Phases CEM I (5 Mass)**			
C_3_S-55.54	C_2_S-14.59	C_3_A-8.15	C_4_AF-6.85

**Table 3:** Chemical properties of cement CEM I 42.5R.

Roasting Loss (%)	Sulfate Content SO_3_ (%)	Chloride Content Cl (%)	Alkali Content Na_2_O_eq_ (%)	SiO_2_ (%)
3.19	2.96	0.05	0.76	20.20
**Al_2_O_3_**	**Fe_2_O_3_**	**CaO**	**CaO_w_**	**MgO**
4.41	2.42	64.36	1.98	1.98

**Table 4:** Concrete mix proportions by weight.

Specification	Mass of Concrete Ingredients (kg/m^3^)			
	Water	Aggregate	Cement	Fly Ash
Ordinary concrete OC	194.88	1807.08	381.94	-
Concrete with quantity 2.5% of fly ash-FA2.5%	194.88	1807.08	372.39	9.54
Concrete with quantity 5% of fly ash-FA5%	194.88	1807.08	362.84	19.10
Concrete with quantity 7.5% of fly ash-FA7.5%	194.88	1807.08	353.29	28.64
Concrete with quantity 10% of fly ash-FA10%	194.88	1807.08	343.74	38.19
Concrete with quantity 12.5% of fly ash-FA12.5%	194.88	1807.08	334.20	47.74
Concrete with quantity 15% of fly ash-FA15%	194.88	1807.08	324.65	57.29
Concrete with quantity 17.5% of fly ash-FA17.5%	194.88	1807.08	315.10	66.84
Concrete with quantity 20% of fly ash-FA20%	194.88	1807.08	305.55	76.39

**Table 5:** Average decrease in compressive strength and average loss in mass of samples subjected to freezing.

Concrete Sample	Average Compressive Strength		Average Strength Decrease of Frozen Samples	Average Strength Decrease of Frozen Samples		Average Loss in Mass
	of Reference Sample (MPa)	after 150 Freeze-Thaw Cycles (MPa)		before Freezing (g)	after 150 Freeze-Thaw Cycles (g)	
OC	51.26	42.26	−17.56	2409	2385	0.996
FA2.5%	44.62	43.42	−2.69	2372	2360	0.506
FA5%	45.36	43.50	−4.10	2411	2386	1.037
FA7.5%	46.97	43.62	−7.13	2380	2358	0.924
FA10%	48.16	43.59	−9.49	2398	2390	0.334
FA12.5%	50.47	43.52	−13.77	2381	2372	0.378
FA15%	52.36	43.90	−16.16	2393	2387	0.251
FA17.5%	55.12	43.66	−20.79	2372	2360	0.506
FA20%	57.25	44.56	−22.69	2399	2387	0.500

**Table 6:** ANOVA table for compressive strength of concrete samples at different temperatures.

Concrete Sample		Sum of Squares	df	Mean Square	F	Significance Value
OC	Between Groups	1548.040	3	516.013	53.008	0.000
FA5%	Between Groups	1537.574	3	512.525	70.081	0.000
FA10%	Between Groups	1658.781	3	552.927	236.395	0.000
FA15%	Between Groups	2034.499	3	678.166	387.360	0.000
FA20%	Between Groups	1267.376	3	422.459	43.008	0.000

**Table 7:** ANOVA table for compressive strength of concrete samples after 150 freeze-thaw cycles.

Concrete Sample		Sum of Squares	df	Mean Square	F	Significance Value
Control	Between Groups	471.830	8	58.979	24.566	0.000
After 150 freeze-thaw	Between Groups	1337.063	8	167.133	27.369	0.000
